# Taxonomic structure of bacterial communities in sourdoughs of spontaneous fermentation

**DOI:** 10.18699/VJGB-22-47

**Published:** 2022-07

**Authors:** V.K. Khlestkin, M.N. Lockachuk, O.A. Savkina, L.I. Kuznetsova, E.N. Pavlovskaya, O.I. Parakhina

**Affiliations:** All-Russian Research Institute of Genetics and Breeding of Farm Animals – Branch of L.K. Ernst Federal Research Center for Animal Husbandry, Pushkin, St. Petersburg, Russia; Saint-Petersburg Brunch of the Scientific Research Institute for the Baking Industry, Pushkin, St. Petersburg, Russia; Saint-Petersburg Brunch of the Scientific Research Institute for the Baking Industry, Pushkin, St. Petersburg, Russia; Saint-Petersburg Brunch of the Scientific Research Institute for the Baking Industry, Pushkin, St. Petersburg, Russia; Saint-Petersburg Brunch of the Scientific Research Institute for the Baking Industry, Pushkin, St. Petersburg, Russia; Saint-Petersburg Brunch of the Scientific Research Institute for the Baking Industry, Pushkin, St. Petersburg, Russia

**Keywords:** rye sourdough, microbiome, microbial community, lactobacillus, high-throughput sequencing, fermentation, bakery products, ржаная закваска, микробиом, микробное сообщество, лактобактерии, высокопроизводительное секвенирование, ферментация, хлебобулочные изделия

## Abstract

The article is devoted to the study of the microbiome of spontaneously fermented sourdoughs. The aim of the work was to study the inf luence of the technological parameters of sourdough propagations on the taxonomic structure of the microbiome of spontaneously fermented sourdoughs. Two spontaneously fermented sourdoughs were studied: dense rye sourdough and liquid rye sourdough, both prepared using the same batch of peeled rye f lour. To study the taxonomic structure of the sourdough microbiome in dynamics, the method of high-throughput sequencing of 16S rRNA gene fragments of microorganisms was used. It was shown that the technological parameters of sourdough (humidity, temperature) do not affect the taxonomic composition of the microbiome of dense rye or liquid rye sourdough at the phylum/class/genus level. It was found that during the f irst three days of propagations, bacteria from the phyla Proteobacteria and Firmicutes dominated in the microbial community. In the phylum Proteobacteria, microorganisms from the order Enterobacterales took a large share, which persisted for three days of backslopping. The phylum Firmicutes was represented by lactic acid bacteria of the genera Weissella, Lactobacillus, Leuconostoc, Pediococcus, Lactococcus. It was established by classical microbiological methods that after a day of fermentation, the number of lactic acid bacteria cells was signif icantly higher in liquid rye sourdough compared to dense one. However, with further propagation of sourdoughs, the number of cells was comparable, while signif icant changes occurred at the level of genera and species. It was shown that as the relative number of lactic acid bacteria of the genus Lactobacillus increased, a gradual displacement of the coccal forms of Lactococcus, Leuconostoc, Weissella, Pediococcus happened. With further propagation of sourdough after 10 days, the position of the dominant groups of bacteria was occupied by representatives of the phylum Firmicutes, lactic acid bacteria of the genus Lactobacillus. The inf luence of the mode and parameters of the sourdough on the species composition of lactobacilli, which demonstrated a low bacterial diversity, is shown. In the f irst three days of propagations, lactobacilli L. curvatus, L. brevis, and Lactiplantibacil- lus sp. dominated in both sourdoughs. After a month of backslopping, Fructilactobacillus sanfranciscensis and Companilactobacillus sp. dominated in dense rye sourdough, and L. pontis dominated in liquid rye sourdough

## Abstract (ru)

Статья посвящена исследованию микробиома хлебных заквасок спонтанного брожения. Цель работы – изучение влияния технологических параметров ведения заквасок на таксономическую структуру микробиома хлебных заквасок спонтанного брожения. Объектами исследования являлись две закваски спонтанного брожения – густая ржаная и жидкая ржаная без заварки, приготовленные с использованием одной партии муки ржаной обдирной. Для изучения таксономической структуры заквасочного микробиома в динамике применяли метод высокопроизводительного секвенирования фрагментов генов 16S рРНК микроорганизмов. Показано, что технологические параметры ведения заквасок (влажность, температура) не оказывают влияния на таксономический состав микробиома густой ржаной и жидкой ржаной заквасок на уровне филумов/классов/родов (но не видов). Установлено, что в течение первых трех суток ведения в микробном сообществе доминировали бактерии из филумов Proteobacteria и Firmicutes. В филуме Proteobacteria большую долю занимали микроорганизмы из порядка Enterobacterales, которые сохранялись в течение трех суток ведения заквасок. Филум Firmicutes был представлен молочнокислыми бактериями родов Weissella, Lactobacillus, Leuconostoc, Pediococcus, Lactococcus. Классическими микробиологическими методами установлено, что через одни сутки брожения количество клеток молочнокислых бактерий было значительно выше в жидкой ржаной закваске по сравнению с густой, однако при дальнейшем ведении заквасок количество клеток было сопоставимым, при этом происходили существенные изменения на уровне родов и видов. Выявлено, что по мере увеличения относительной численности молочнокислых бактерий рода Lactobacillus происходило постепенное вытеснение кокковых форм Lactococcus, Leuconostoc, Weissella, Pediococcus. При дальнейшем ведении заквасок через 10 суток положение доминирующих групп бактерий занимали представители филума Firmicutes – молочнокислые бактерии рода Lactobacillus. Показано влияние режима и параметров ведения заквасок на видовой состав лактобацилл, который демонстрировал низкое бактериальное разнообразие. В первые трое суток ведения в обеих заквасках доминировали лактобациллы L. curvatus, L. brevis и Lactiplantibacillus sp. Через месяц ведения в густой ржаной закваске доминировали Fructilactobacillus sanfranciscensis и Companilactobacillus sp., а в жидкой ржаной – L. pontis.

## Introduction

In recent years, an increased interest in the development of sourdough bakery products, including in handicraft and home conditions, has been observed. This is due to the fact that bakery products on sourdough are characterized by improved taste, aroma, nutritional value and resistance to microbial spoilage. Sourdough is a semi-finished bakery product obtained by fermentation of a nutrient mixture of flour and water by lactic acid bacteria or lactic acid bacteria and baking yeast, which can enter the starter from the feedstock or from industrial starter microbial compositions (Auerman, 2009; De Vuyst et al., 2017).

Sourdough maintenance is a technological process that includes regular refreshing of the sourdough with a portion of flour and water, followed by fermentation until ready. After fermentation, part of the ripe sourdough goes to kneading the dough, and part is used for a new refreshment. This ensures a continuous process of fermentation. This makes it possible to maintain the starter microbiota in an active state and obtain a sourdough with the specified biotechnological and physicochemical parameters, which ensures the production of bakery products with the required consumer properties (Kosovan, 2008; Auerman, 2009).

We have previously shown for the first time (Lokachuk et al., 2020) that during the long-term management of national sourdoughs bred using starting microbial compositions, significant changes in the species diversity of lactobacilli occur, leading to predominance of species other than those introduced in the first phase of the breeding cycle, and, nevertheless, allowing to obtain bakery products corresponding to GOST 2077-84. Thus, we have established one of the reasons why consumers may notice a difference in the physicochemical and organoleptic characteristics of products of the same type, since changes in the microbiome during the long-term management of thick rye sourdough lead to a significant change in the content of lactic and acetic acids, in titrated acidity, lifting force, alcohol content in the starter and dough, and hence, the finished product. Samples of rye bread prepared with a long-term starter culture were characterized by higher acidity, lower alcohol content and a large amount of volatile acids (mainly acetic acid).

At the same time, much attention is paid worldwide to the study of the microbiome of spontaneous sourdoughs, in which the initial microflora of raw materials develops. At the same time, the microbiome of domestic starter cultures of spontaneous fermentation and its changes during technological processes remain unexplored, despite its crucial role in shaping the quality and safety of bakery products.

Currently, the use of high-performance sequencing of the 16S rRNA gene makes it possible to expand our knowledge about the taxonomic structure of the starter culture microbiome. This is of great importance, since a large number of factors influence the diversity and structure of starter microbial communities.

Despite the instability of quality and non-sterility of raw materials, starter cultures are stable ecosystems, which may be due to metabolic adaptations in the starter ecosystem (Müller et al., 2001; Minervini et al., 2012; Viiard et al., 2016).

In the fermentation process of sourdoughs, temperature acts as the main factor affecting the dynamics of the microbial community and the kinetics of metabolite production. The fermentation temperature affects the fermentation coefficient, which is the ratio of the concentrations of lactic and acetic acids. Higher temperatures cause a shift towards an increase in the lactic acid content, thereby increasing the acidity of the starter. Homofermentative and facultatively heterofermentative species of lactic acid bacteria (LAB), belonging, for example, to the group Lactobacillus delbrueckii and often prevailing in sourdoughs managed at elevated temperatures, cause a rapid decrease in the pH of the water-flour nutrient mixture mainly due to the formation of lactic acid. Heterofermentative types of LAB, as a rule, predominate in sourdoughs that are managed at lower temperatures and during long periods of fermentation, produce a mixture of lactic, acetic acids and/or ethanol (De Vuyst et al., 2017).

There is a positive correlation between the fermentation temperature (< 30 °C) and the frequency of detection of L. san-franciscensis in sourdoughs. On the contrary, such temperatures negatively correlate with the presence of L. fermentum and L. plantarum species in the sourdough. For example, it has been shown that L. sanfranciscensis prevails in traditional renewable thick sourdoughs, which are managed at temperatures below 30 °C and have pH of about 4 – type I sourdoughs according to European classification (Bӧcker et al., 1995; Hammes et al., 2005) – being optimally associated with yeast C. humilis at a temperature of 25–30 °C (Van Kerrebroeck et al., 2017). Since C. humilis has a temperature optimum of 27–28 °C and cannot grow at temperatures above 35 °C, elevated temperatures negatively affect this mutualistic symbiosis. L. sanfranciscensis is also uncompetitive at elevated temperatures (Gänzle et al., 1998; Vogelmann, Hertel, 2011) compared to other types of LAB.

Humidity and pH also have a significant effect on microbial diversity in sourdoughs. Low pH values stimulate the development of acid-resistant lactobacilli, while higher pH values are favorable for Enterococcus, Lactococcus, Leuconostoc, Pediococcus and Weissella species (De Vuyst et al., 2017). For example, relatively high pH values, usually exceeding 4.0, in type I French sourdoughs may explain the detection of acid-sensitive species P. pentosaceus, Leuconostoc and Weissella (Robert et al., 2009).

It is known that the acidity of the sourdough affects the content of the specie L. sanfranciscensis, the optimal pH value for the growth of which is 5.0. Nevertheless, this type of LAB demonstrates adaptation to acid stress, like many other strains of LAB isolated from sourdoughs.

The L. sanfranciscensis species is mainly found in type I sourdoughs with low humidity and is displaced in sourdoughs with too low a pH value, which persists for a significant period of time during the fermentation, since this type of lactobacilli does not grow at a pH below 3.8. On the contrary, it has been found that acid-resistant species of LAB, such as L. fermentum, L. plantarum, L. reuteri, L. rossiae and/or L. pontis, predominate in liquid non-renewable sourdoughs, which are derived using starting microbial compositions, and are characterized by a higher maintenance temperature (above 30 °C) and prolonged fermentation from 15 h to 3 days – type II sourdoughs (Meroth et al., 2003; Vogelmann, Hertel, 2011; De Vuyst et al., 2017).

Dried or stabilized liquid sourdoughs (type III) contain LAB, which are resistant to the drying process and are able to maintain viability in this form, for example, obligately heterofermentative L. brevis, facultatively heterofermentative L. plantarum, P. pentosaceus (De Vuyst, Neysens, 2005; Hammes et al., 2005; Settanni et al., 2013).

The above data suggest that significant differences in the microbiome and, in particular, in the species composition of lactobacilli can also be detected in domestic sourdoughs with different humidity and maintenance temperature.

For many decades, five types of rye sourdoughs have been widely used at baking plants in Russia for the production of rye and rye-wheat bread: thick sourdough, liquid sourdough without welding, liquid sourdough with welding, concentrated lactic acid – enriched sourdough and thermophilic sourdough. According to Scientific Research Institute for the Baking Industry (Kuznetsova et al., 2021), thick rye sourdough and liquid one without welding are most often used. Both sourdoughs are used in the production of bakery products from a mixture of rye and wheat flour with full or partial replacement of pressed yeast and allow to get high-quality bread.

The choice of a particular sourdough at the plant is determined by technological capabilities (equipment, operating mode). Liquid sourdoughs are used in plants designed to transport the sourdough through pipes from the starter shop to the place where the dough is kneaded, and having tanks with a jacket to ensure the desired temperature. To prepare such a sourdough, a nutritious mixture of flour and water with a moisture content of 70–75 % is used, the fermentation temperature is 30–32 °C. Thick rye sourdough differs significantly in technological parameters: it has a moisture content of 48–50 % and a fermentation temperature of 26–28 °C. It is easier to preserve it during breaks in work, and it does not require the use of tanks with a jacket for fermentation, since the temperature in the workshop conditions is sufficient (Kosovan, 2008). A higher fermentation temperature of the liquid sourdough allows to achieve the necessary technological parameters (acidity and lifting force) in a shorter time, which speeds up the technological process. Given the significant difference in the parameters of the sourdoughs, it is of great interest to study the differences in the formation of their microbiome.

The aim of the work was to study the influence of technological parameters of sourdoughs on the taxonomic structure of the microbiome of bread sourdoughs of spontaneous fermentation.

## Materials and methods

Preparation and maintenance of sourdoughs. Two sourdoughs of spontaneous fermentation were studied: thick rye sourdough and liquid rye sourdough without welding, derived using one batch of rye flour (OAO Kirov LKHP, Russia). Flour quality indicators: the drop number is 193 s, the moisture content is 12.4 %. The moisture content of flour was determined according to GOST 9404-88, the drop number – according to GOST 27676-88. The experiment was carried out in two repetitions.

Lactic acid bacteria of the genera Lactobacillus, Weissella, Pediococcus and Leuconostoc were detected in rye flour, studied in the form of a water-flour nutrient mixture for sourdough. Genus Lactobacillus was the dominant. Lactobacilli of the species F. sanfranciscensis, L. pontis, L. brevis, L. plantarum, Companilactobacillus sp., L. curvatus were also found.

The sourdoughs were managed under laboratory conditions for a month. Nutritional mixtures with a moisture content of 50 and 70 % by weight of 1000 g each were prepared from the same batch of rye flour. To do this, flour and water were mixed to a homogeneous consistency in a ratio of 1:0.76 and 1:1.9, respectively.

The obtained water-flour nutrient mixtures with a moisture content of 50 and 70 % were left to ferment in thermostats for two days at temperatures of 26 ± 1 °С and 32 ± 1 °С , respectively.

The fermented sourdoughs were updated with appropriate nutrient mixtures in the ratio of sourdough: the nutrition 1:1 and left for another day of fermentation at temperatures of 26 ± 1 °С and 32 ± 1 °С, depending on the moisture content (50 or 70 %) of the starter.

During the following days, the sourdoughs were renewed in a ratio of 1:1 after 6.5–7 h and 16 h and fermented at appropriate temperatures. After 16-h fermentation, the sourdoughs were renewed in a ratio of 1:1, each sourdough was fermented for 3.5–4 h at a given temperature, tested for quality (lifting force, titrated acidity, volume increase in % to the original volume) and sent for storage in the refrigerator at a temperature of 5 ± 1 °С for 2.5 days. The acidity was determined by titration with 0.1 N sodium hydroxide solution in the presence of phenolphthalein and presented in degrees. The lifting force was determined by the “ball” method and expressed in minutes (Puchkova, 2004).

Over the next four weeks, from Monday to Thursday, the sourdoughs with a moisture content of 50 % were refreshed in the ratio sourdough: the nutrient mixture 1:3 and fermented at a temperature of 26 °С for 7 h, and then refreshed in a ratio of 1:5 at a temperature of 20 °С and left for 16 h at a temperature of 17–18 °С. During the same period, the sourdoughs with a moisture content of 70 % were refreshed in a ratio of 1:2 with a temperature of 32 °С and fermented for 7 h. Then they were refreshed in the same ratio, but with a temperature of about 20 °C and left to ferment for 16 h at a temperature of 17–18 °С. On Friday, the sourdoughs were updated according to the regime established for each sourdough, fermented for 3.5–4 h at a given temperature and after quality control were placed in the refrigerator and stored at a temperature of 4–5 °С for 2.5 days.

These two temperature and humidity regimes are actually used at the plants in Russia due to technical reasons. So, for a thick rye sourdough, it is technically impossible to provide a higher fermentation temperature, since it is impossible to unload from a container with a jacket, and transportation of the sourdough through a pipeline is impossible. It is possible to manage such a sourdough only under those regimes that are installed at plants

The objective of this study was to identify the influence of these modes and parameters characteristic of our industry on the microbiome of starter cultures

Microbiological analysis of sourdoughs. The number of viable cells of lactic acid bacteria was controlled during the management of sourdoughs. For that, 10 g of the sourdough sample was homogenized manually in a mortar in 90 ml of 0.9 % sodium chloride solution. A series of tenfold consecutive dilutions was prepared and seeded on Sanfrancisco agar (Picozzi et al., 2005). To create anaerobic cultivation conditions, gas packages (AnaeroGen) were used, providing a carbon dioxide level of 9 to 13 % and an oxygen content of less than 1 %. The crops were incubated at 30 °С.

Determination of the composition of microbial communities by high-throughput sequencing of a fragment of the 16S rRNA gene. In each sample, the taxonomic structure of the microbiome was determined by high-performance sequencing based on the Collective Use Center of “Genomic Technologies, Proteomics and Cell Biology” of the Federal State Budget Scientific Institution All-Russia Research Institute for Agricultural Microbiology

To isolate DNA from the samples, a set of reagents (MACHEREY- NAGEL NucleoSpin Soil) from MACHEREYNAGEL (Germany) was used according to the manufacturer’s instructions. The taxonomic composition of the bacterial community was determined in each sample based on the analysis of amplicon libraries of ribosomal operon fragments. The taxonomic analysis of the bacterial community was performed with universal primers F515/R806 for a variable region of the 16SpRNA-v4 gene (GTGCCAGCMGCCGCGGTAA/ GGACTACVSGGGTATCTAAT) specific for a wide range of microorganisms, including bacteria and archaea (Bates et al., 2010). All primers included service sequences containing linkers and barcodes (necessary for sequencing using Illumina technology).

PCR was performed in 15 μl of a reaction mixture containing 0.5–1 unit of activity of Q5® High-Fidelity DNA Polymerase polymerase (NEB, USA), 5 pcM of direct and reverse primers, 10 ng of DNA matrix and 2nM of each dNTP (LifeTechnologies, USA). The mixture was denatured at 94 °С for 1 min, followed by 35 cycles: 94 °С – 30 s, 50 °С – 30 s, 72 °С – 30 s. The final elongation was carried out at 72 °C for 3 min. PCR products were purified according to the Illumina recommended method using AMPureXP (BeckmanCoulter, USA). Further preparation of libraries was carried out in accordance with the instructions of the manufacturer MiSeq Reagent Kit Preparation Guide (Illumina, USA). The libraries were sequenced in accordance with the manufacturer’s instructions on the Illumina MiSeq device (Illumina, USA) using the MiSeq® ReagentKit v3 reagent kit (600 cycles) with two-way reading (2*300 n).

The data obtained as a result of sequencing samples were processed using software packages Trimomatic (Bolger et al., 2014) and QIIME (Caporaso et al., 2010). At the first stage, the primary analysis of the reading quality, the selection of sequences based on the reading quality of individual bases (base pair quality), the combination of paired-terminal sequences with an overlap area of at least 35 bases was performed, as well as the removal of sequences the length of which is less than 180 bp. At the second stage of processing, all service sections were removed from libraries (primers), as well as sequences containing extended homopolymer repeats. De novo OTE-picking was used in the analysis of bacterial communities. Taxonomic identification of OTE was carried out using the RDP database (http://rdp.cme.msu.edu).

## Results

Lactic acid bacteria of the genera Lactobacillus, Weissella, Pediococcus and Leuconostoc, characteristic for the natural seeding of grain and flour, were detected in rye flour, studied in the form of a water-flour nutrient mixture for starter culture. Genus Lactobacillus was the dominant. Lactobacilli of the species F. sanfranciscensis, L. pontis, L. brevis, L. plantarum, Companilactobacillus sp., L. curvatus were also found.

**Fig. 1.**
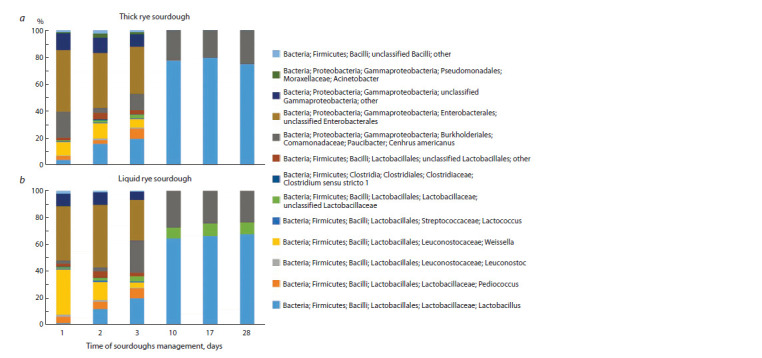
Composition of microbial communities according to the analysis of sequences of 16S rRNA gene fragments in spontaneous sourdoughs: dense rye sourdough (a), liquid rye sourdough (b).

According to the data obtained, during the first three days of management, the bacterial complex of the studied sourdough microbiomes was formed by representatives of two phyla: Proteobacteria (the Gammaproteobacteria class dominated) and Firmicutes (lactic acid bacteria of the genera Weissella, Lactobacillus, Leuconostoc, Pediococcus, Lactococcus). The phylum Protrobacteria was represented by bacteria of the order Enterobacterales (about 40 %), which after 48 h of fermentation were also detected in significant quantities (Fig. 1).

On the third day of sourdough management, the number of bacteria of the order Enterobacterales decreased slightly to 30 %. It is worth noting that during this period the sourdoughs had an unpleasant putrid smell. With further management of sourdoughs after 10 days, representatives of the phylum Firmicutes dominated lactic acid bacteria of the genus Lactobacillus, extraneous bacteria of the order Enterobacterales were not detected. Both sourdoughs acquired a characteristic sourdough smell at that time.

**Fig. 2.**
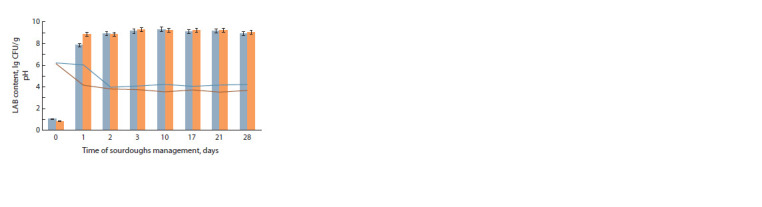
Changes in number of lactic acid bacteria cells and pH in sourdoughs during 28 days

Control of the number of viable LAB cells by classical microbiological methods showed that after a day of fermentation, the number of LAB cells was significantly higher in liquid rye sourdough. This may be explained by a higher temperature of 32 °С compared to a thick sourdough, which was conducted at a temperature of 26 °С. With further management of sourdoughs, the number of LAB cells was comparable, but significant changes occurred at the level of genera and species (Fig. 2).

**Fig. 3.**
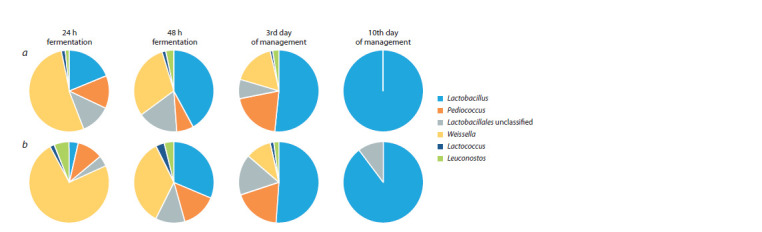
Representation of LAB genera (%) according to the analysis of sequences of 16S rRNA gene fragments in: dense rye sourdough (a); liquid rye sourdough (b).

Lactic acid bacteria of the genus Weissella dominated in both sourdoughs after a day of fermentation (53 % of the total amount of LAB in thick and 74 % in liquid sourdough), LAB of the genera Lactobacillus, Leuconostoc, Pediococcus, Lactococcus were also detected (Fig. 3). At the same time, an extremely low content of the most significant for sourdoughs genus Lactobacillus was noted in the liquid sourdough (3 %). With further management of sourdoughs, as the number of LAB of the genus Lactobacillus increased, there was a gradual displacement of coccoid forms of Lactococcus, Leuconostoc, Weissella, Pediococcus. After three days, the number of lactobacilli increased in both sourdoughs to 50 %. On the 10th day of management, only LAB of the genus Lactobacillus were detected.

It was found that during the management of sourdoughs, significant changes occurred not only at the level of LAB genera, but also at the level of lactobacillus species.

After 24 h of fermentation, Lactobacillus curvatus dominated in both sourdoughs. The microbiota after 48 h of fermentation in both sourdoughs was represented by lactobacilli Latilactobacillus curvatus, Levilactobacillus brevis and Lactiplantibacillus plantarum/paraplantarum/pentosus/fabifermentans (could not be accurately identified due to the low difference in the nucleotide sequences of the 16S rRNA gene in these species), which were found in the initial water-flour nutrient mixture before fermentation. With further management of sourdoughs, the content of these genera decreased.

After 10 days of management and throughout the rest of the period, obligately heterofermentative lactobacilli of the species Fructilactobacillus sanfranciscensis, also found in rye flour, dominated the thick rye sourdough. After a month of sourdough management, a species belonging to Companilactobacillus sp. was identified, which could not be uniquely identified using the RDP database. The source of this type of LAB was also rye flour. During the first two weeks of the sourdough management this type was present in the sourdough in small quantities 

Obligately heterofermentative LAB of the species Limosilactobacillus pontis prevailed in the liquid rye sourdough without welding, and were also found in the flour sample.

**Fig. 4.**
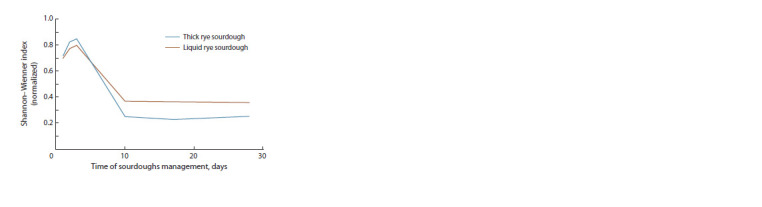
Biodiversity change in the process of the rye sourdoughs

The alpha diversity was evaluated (Fig. 4). The Shannon– Wiener index was calculated using the formula

**Formula:**
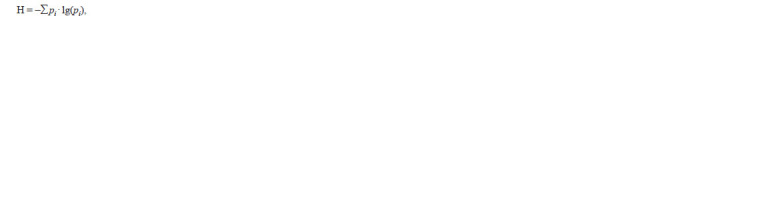
Formula

where i is the number of lactobacillus species found in the starter culture, pi is the proportion occupied by a particular species in the total population of lactobacillus species.

The obtained results fully confirm the fact that the parameters of sourdoughs (temperature, humidity) significantly affect the species diversity of lactobacilli, which was low in both sourdoughs.

## Discussion

In this paper, for the first time, the influence of technological parameters of sourdoughs on the taxonomic structure of the microbiome of domestic bread sourdoughs of spontaneous fermentation was studied. Using a culture-independent method of high-performance sequencing of the 16S rRNA gene, it was shown that bacteria belonging to two phyla: Firmicutes and Proteobacteria were detected in the sourdoughs during the first three days. The relative number of proteobacteria decreased during fermentation and after 10 days of management they were not detected. This is completely consistent with previous results of other researchers showing that several phyla of bacteria in addition to Firmicutes (for example, Actinobacteria, Bacteroidetes, Cyanobacteria and Proteobacteria) may be present in the sourdoughs before fermentation begins. Most of them are inactive populations and are completely displaced by Firmicutes (Ercolini et al., 2013; Rizzello et al., 2015; Menezes et al., 2020).

At the same time, the source of extraneous microbiota is raw materials. Typically, the bacterial population of rye and wheat flour that does not belong to the phylum Firmicutes consists of representatives of the phylum Proteobacteria (for example, the genus Erwinia, Acinetobacter, Pantoea, Pseudomonas, Comamonas, Enterobacter and Sphingomonas) and Bacteroidetes (for example, Chryseobacterium). This population is usually almost completely suppressed on the first day of sourdough management. The exception is representatives of the Enterobacteriaceae family, which are detected up to 5 days of sourdough, probably due to the ability to synthesize organic acids and some resistance to acid stress (Ercolini et al., 2013). Thus, during fermentation, microbial diversity changes with an increase in the proportion of the phylum Firmicutes and the displacement of representatives of the phylum Proteobacteria.

The phylum Firmicutes was represented by lactic acid bacteria. It was found that on the first day, representatives of the genus Weissella dominated in both sourdoughs after 24 h of fermentation. The bacteria of the genus Lactobacillus, Leuconostoc, Pediococcus, Lactococcus were contained in smaller quantities, and after three days the number of LAB of the genus Lactobacillus increased in both sourdoughs to 50 % of the total number of LAB, and coccoid forms of Lactococcus, Leuconostoc, Weissella, Pediococcus were displaced. On the 10th day of management, only LAB of the genus Lactobacillus were detected. In the study of Polish rye starter cultures, a similar dynamics was noted (Boreczek et al., 2020): after 24 h of fermentation in the sourdough, the content of bacteria of the genus Weissella was 36 %, and after 72 h – only 5 %, while the content of the genus Lactobacillus increased from 30 to 67 % by the third day of the sourdough management.

The data obtained differ somewhat from the data obtained for Italian sourdoughs, in which, after 10 days, the content of representatives of the genus Weissella was almost 2 times greater than that of the genus Lactobacillus (Ercolini et al., 2013). The authors suggest that this is due to the fact that the genus Weissella dominated in Italian rye flour. Indeed, Lactococcus, Enterococcus, Leuconostoc and Weissella are usually found in grain and flour, respectively, but are not able to withstand a long acidification process, since their development requires higher pH values compared to lactobacilli (Van Kerrebroeck et al., 2017). A number of studies have shown that the growth of representatives of the genus Weissella is inhibited at pH 4.5, but they are capable of growth at pH 6.5– 6.8. The acidic environment of the sourdough stimulates the change of LAB communities in the sourdoughs and creates a niche favorable for the development of acid-resistant lactobacilli, such as L. brevis and L. sanfranciscensis (Oshiro et al., 2020).

Summarizing the known and obtained data, it can be noted that the stabilization of the microbiome with the predominance of the genus Lactobacillus occurs by 10 days (Van der Meulen et al., 2007; Weckx et al., 2010). This correlates with our data on the change in alpha diversity (see Fig. 4) of thick and liquid rye sourdoughs

It is noted that during the same period there are significant changes in the species composition of lactobacilli. The dominant species after 24 h of fermentation, Latilactobacillus curvatus, was discovered after 48 h together with the species Levilactobacillus brevis and Lactiplantibacillus plantarum/ paraplantarum/pentosus/fabifermentans, which were found in small quantities and in the initial water-flour nutrient mixture before fermentation

A decrease in the content of Latilactobacillus curvatus in the process of sourdoughs was also noted by foreign researchers. It was found that at the first stages of the management of Korean sourdoughs, the content of certain types of LAB was: L. curvatus (9.5 log CFU/g), F. sanfranciscensis (< 5 log CFU/g), L. brevis (6.5 log CFU/g), whereas after the 11th refreshment, the number of F. sanfranciscensis significantly increased (> 9.0 log CFU/g), while the content of L. curvatus and L. brevis decreased, which, according to the authors, is due to the negative effect of increased lactic acid content in the medium on the development of L. curvatus (Baek et al., 2021). Studies (Landis et al., 2021) have shown that L. plantarum and L. brevis were the most frequently detected pair of simultaneously occurring taxa (in 177 out of 500 sourdoughs), while the species L. sanfranciscensis dominated in most long-term sourdoughs, and its content negatively correlated with the content of L. plantarum and L. brevis species, which dominated in young sourdoughs

With further management of sourdoughs, significant differences were noted in the formation of microbiota in liquid and thick rye sourdoughs. In thick rye sourdough, an increase in the content of bacteria of the species Fructilactobacillus sanfranciscensis was noted. According to the literature data, this species is considered the most adapted and is an autochthonous microorganism of the type I sourdough microbiota (Siragusa et al., 2009; Vogel et al., 2011; Rogalski et al., 2020). The dominance of this species in thick rye sourdough is explained by the creation of optimal conditions for its development (temperature less than 30 °C, pH within 4.1–4.3, moisture content 50 %). However, after a month of keeping in the sourdough in addition to F. sanfranciscensis lactic acid bacteria Companilactobacillus sp. were found, the source of which was flour.

Obligately heterofermentative LAB of the species Limosilactobacillus pontis found in liquid rye sourdough without welding developed at higher temperatures (32 °С) and moisture content (70 %) and lower pH (3.6–4.0). It is obvious that these conditions are favorable for the development of this species, which is confirmed by the literature data (De Vuyst et al., 2017).

## Conclusion

As a result of the research, the diversity of prokaryotes in domestic sourdoughs of spontaneous fermentation was studied for the first time by the method of high-performance sequencing. It was found that during the first three days of management, the bacterial complex of the studied sourdoughs was represented by the phyla Proteobacteria (class Gammaproteobacteria) and Firmicutes (lactic acid bacteria of the genera Weissella, Lactobacillus, Leuconostoc, Pediococcus, Lactococcus). In the further management of sourdoughs after 10 days, representatives of the phylum Firmicutes dominated lactic acid bacteria of the genus Lactobacillus

A comparative analysis of the taxonomic composition of the microbiome of thick and liquid rye sourdough without welding did not demonstrate deep differences throughout the entire period of sourdough management both at the phylum/class level and at the generic level. However, there was a difference at the level of lactobacilli species, which is due to the influence of exogenous factors, such as temperature and humidity of sourdoughs, on the formation of the starter microbiome

Further studies of industrial and laboratory sourdoughs of long-term management will allow us to establish whether there is a stabilization of the microbiome with the dominance of one or two species, whether periodic fluctuations in the composition of the microbiome occur, or other scenarios are being implemented.

## Conflict of interest

The authors declare no conflict of interest.
